# Epic Immune Battles of History: Neutrophils vs. *Staphylococcus aureus*

**DOI:** 10.3389/fcimb.2017.00286

**Published:** 2017-06-30

**Authors:** Fermin E. Guerra, Timothy R. Borgogna, Delisha M. Patel, Eli W. Sward, Jovanka M. Voyich

**Affiliations:** Department of Microbiology and Immunology, Montana State UniversityBozeman, MT, United States

**Keywords:** host-pathogen interactions, innate immunity, immune evasion, host defense, phagocytosis, chemotaxis, *Staphylococcus aureus*

## Abstract

Neutrophils are the most abundant leukocytes in human blood and the first line of defense after bacteria have breached the epithelial barriers. After migration to a site of infection, neutrophils engage and expose invading microorganisms to antimicrobial peptides and proteins, as well as reactive oxygen species, as part of their bactericidal arsenal. Ideally, neutrophils ingest bacteria to prevent damage to surrounding cells and tissues, kill invading microorganisms with antimicrobial mechanisms, undergo programmed cell death to minimize inflammation, and are cleared away by macrophages. *Staphylococcus aureus* (*S. aureus*) is a prevalent Gram-positive bacterium that is a common commensal and causes a wide range of diseases from skin infections to endocarditis. Since its discovery, *S. aureus* has been a formidable neutrophil foe that has challenged the efficacy of this professional assassin. Indeed, proper clearance of *S. aureus* by neutrophils is essential to positive infection outcome, and *S. aureus* has developed mechanisms to evade neutrophil killing. Herein, we will review mechanisms used by *S. aureus* to modulate and evade neutrophil bactericidal mechanisms including priming, activation, chemotaxis, production of reactive oxygen species, and resolution of infection. We will also highlight how *S. aureus* uses sensory/regulatory systems to tailor production of virulence factors specifically to the triggering signal, e.g., neutrophils and defensins. To conclude, we will provide an overview of therapeutic approaches that may potentially enhance neutrophil antimicrobial functions.

## Introduction

Polymorphonuclear leukocytes (PMNs or neutrophils) are the first line of defense against bacterial pathogens that have breached epithelial barriers. Within minutes of bacterial invasion, neutrophils respond to soluble factors including chemokines and cytokines and are recruited to the site of infection where they ingest microbes. Subsequently, neutrophils expose microorganisms to antimicrobial proteins, peptides, and reactive oxygen species to kill the invading pathogen. This is a delicate process that must eliminate the pathogen while controlling excessive inflammation. Concurrently, neutrophils secrete cytokines and chemokines to continue to recruit neutrophils and enhance other host responses to infection. Finally, neutrophil death is essential for proper resolution of infection and must be regulated to minimize bystander damage while continuing to signal if more immune response is needed or if tissue repair should begin. These potent mechanisms are effective at eliminating most fungal and bacterial microorganisms. However, successful pathogens have developed strategies to disrupt various neutrophil functions to cause infection.

*S. aureus* is a highly-adaptable Gram-positive pathogen estimated to colonize 50–60% of the population (Wertheim et al., 2005; Gorwitz et al., 2008). It is also a leading cause of infections ranging from superficial skin abscesses to life-threatening diseases, including septicemia and necrotizing pneumonia (Klevens et al., 2007; Kobayashi et al., 2015). The ability of *S. aureus* to cause human disease is based in part on its ability to evade the innate immune response, thereby circumventing rapid elimination. Many factors contribute to *S. aureus* pathogenesis. These include production of numerous toxins, such as the barrel forming two-component toxins capable of directly lysing host immune cells (Menestrina et al., 2003), and tissue destroying enzymes including protease, lipase, and hyaluronidase, as well as many surface proteins and adhesins linked to virulence (Lowy, 1998). In this review, we will focus on evasion strategies used by *S. aureus* to disrupt neutrophil functions essential for bacterial clearance. First, we will highlight virulence factors produced by *S. aureus* to alter neutrophil priming, activation, chemotaxis, and adhesion. Then, we will discuss strategies used by *S. aureus* to subvert neutrophil killing by antimicrobial peptides and proteins and reactive oxygen species. Additionally, we will examine recent literature investigating mechanisms used by *S. aureus* to modulate neutrophil cell death programs. Finally, we will highlight the reciprocal communication between *S. aureus* and the neutrophil emphasizing sensing and adaptive responses used by *S. aureus* to recognize and respond to neutrophil challenge. The review will conclude with an overview of potential therapeutic approaches aimed at disrupting bacterial sensing and signaling to decrease production of virulence factors during neutrophil interaction and discuss putative immunotherapies to boost immune responses to *S. aureus* while limiting inflammatory damage caused by neutrophils.

## Strategies used by *S. aureus* to disrupt neutrophil priming, activation, chemotaxis and adhesion

Neutrophils are initially recruited to a site of infection by following chemokine gradients in a process termed chemotaxis. Taking cues from activated endothelium, neutrophils slow their movement through blood vessels by selectin-mediated tethering to the endothelium followed by complete movement arrest through interaction with integrins on the endothelium. Extravasation from the blood vessels through the endothelial barrier is required for neutrophils to access interstitial fluid and migrate via a chemotactic gradient to the site of infection where ingestion of bacteria can take place. For detailed reviews of neutrophil chemotaxis, adhesion to the epithelium, and transmigration, please refer to (Kolaczkowska and Kubes, 2013; de Oliveira et al., 2016). Herein, we will focus on virulence factors produced by *S. aureus* to inhibit specific neutrophil receptors from binding host and bacterial derived ligands, which results in impaired neutrophil priming, activation, chemotaxis, and adhesion to the endothelium.

## Neutrophil priming: a potential target of *S. aureus*?

Priming refers to the ability of a primary agonist to enhance a neutrophil's response to a secondary stimulus (Swain et al., 2002). There are many known neutrophil priming agents including: complement components C3a and C5a (Skjeflo et al., 2014), interferon-γ (IFN-γ) (Edwards et al., 1988), interleukin-8 (IL-8) (Mitchell et al., 2003), and tumor-necrosis factor-α (TNF-α; Rainard et al., 2000). Bacterial derived products such as N-formyl methionyl peptide, formyl-methionyl-leucyl phenylalanine (fMLF), peptidoglycan, and *S. aureus* cytolytic toxins also demonstrate an ability to prime neutrophils (Elbim et al., 1994; El-Benna et al., 2008; Clarke et al., 2010; Malachowa et al., 2012). The ability of these agents to prime neutrophils is typically not universal in that concentration and neutrophil response can vary drastically (Swain et al., 2002). Primed neutrophil responses influence many neutrophil functions including increases in adhesion, phagocytosis, superoxide production, and degranulation (Ellis and Beaman, 2004). It can also influence neutrophil apoptosis (Wright et al., 2013). Thus, priming can set the stage for subsequent neutrophil-pathogen interactions and influences outcome of this interaction.

Not much is known about the impact of *S. aureus* on neutrophil priming. Earlier studies investigated priming of neutrophils with conditioned medium from peripheral blood mononuclear cells (PBMC) challenged with killed *S. aureus* (Ferrante et al., 1989; Bates et al., 1991). These studies demonstrated increased neutrophil staphylocidal activity following priming with conditioned media from PBMCs challenged with killed *S. aureus* vs. priming with medium from unstimulated PBMCs. This enhanced bactericidal activity was shown to be dependent on TNF-α produced by PBMCs in response to killed *S. aureus* (Ferrante et al., 1989, 1993; Bates et al., 1991). These studies suggest that the response of PBMCs and resident cells to *S. aureus* may strongly impact the outcome of neutrophil-*S. aureus* interactions. Supporting this idea are studies demonstrating that *S. aureus* promotes production of IFN-γ and this production has been linked to poorer outcome of infection in mouse models (Watkins et al., 2011, 2013). IFN-γ impacts neutrophil function in many ways, including priming of oxidative burst and degranulation mechanisms (Ellis and Beaman, 2004). Using a murine wound model of *S. aureus* infection, IFN-γ-mediated CXC chemokine production by T cells promoted a robust recruitment of neutrophils that resulted in elevated *S. aureus* burdens at the infectious foci (McLoughlin et al., 2008). Subsequent studies using a mouse peritonitis model also demonstrated IFN-γ was associated with higher bacterial burdens but that the source of IFN-γ was neutrophils (Watkins et al., 2013). Potentially, priming by IFN-γ causes overactivation and inflammation in neutrophils making them less effective at clearing *S. aureus*. The role of IFN-γ and neutrophil bactericidal activity against *S. aureus* warrants further investigation especially considering that IFN-γ is used to treat patients with chronic granulomatous disease (Gallin et al., 1991). CGD patients receiving recombinant IFN-γ show a decrease in *S. aureus* infections (Gallin et al., 1991). Furthermore, *in vitro* studies have demonstrated that priming of human neutrophils with IFN-γ increased bactericidal activity against *S. aureus* (Edwards et al., 1988). Thus, more studies are needed to determine under what conditions IFN-γ promotes an effective neutrophil response against clinically relevant strains of *S. aureus*. Clearly, source, timing and amount of IFN-γ play an important role in outcome of *S. aureus*-neutrophil interactions.

*S. aureus* produces hemolysins, and bi-component leukocidins that directly impact immune cell function (Seilie and Bubeck Wardenburg, 2017). Many of these toxins act by forming pores in the membrane of immune cells causing lysis. Among these, the bi-component toxins leukotoxin GH (LukGH, also known as LukAB) and Panton-Valentine leukocidin (PVL) have the ability to cause neutrophil pore-formation when present at high concentrations. However, at sublytic concentrations, these factors can promote enhanced binding, uptake and killing of *S. aureus* through priming of neutrophils (Graves et al., 2012). This is also consistent with reports that have demonstrated the ability of PVL to enhance neutrophil superoxide, granule exocytosis, and release of leukotriene B_4_ and IL-8 (König et al., 1995; Colin and Monteil, 2003). In addition, low doses of alpha-toxin, a single component toxin encoded by *hla*, has been shown to promote transcriptional activity leading to production of inflammatory mediators that can prime neutrophils including IL-8 (Dragneva et al., 2001).

As mentioned above, most of the studies thus far have not directly investigated how modulation of known neutrophil priming agents (such as cytokines and chemokines) by *S. aureus* actually influences neutrophil function. Also, the impact of *S. aureus* toxins and immunomodulatory proteins on neutrophil priming has not been thoroughly investigated. Taken together, targeting neutrophil priming may be very important for *S. aureus* to gain an edge on evading the neutrophil. However, additional studies are needed to understand the impact of *S. aureus* on neutrophil priming.

## Key factors used by *S. aureus* to disrupt neutrophil activation, chemotaxis and adhesion

**Figure 1:**
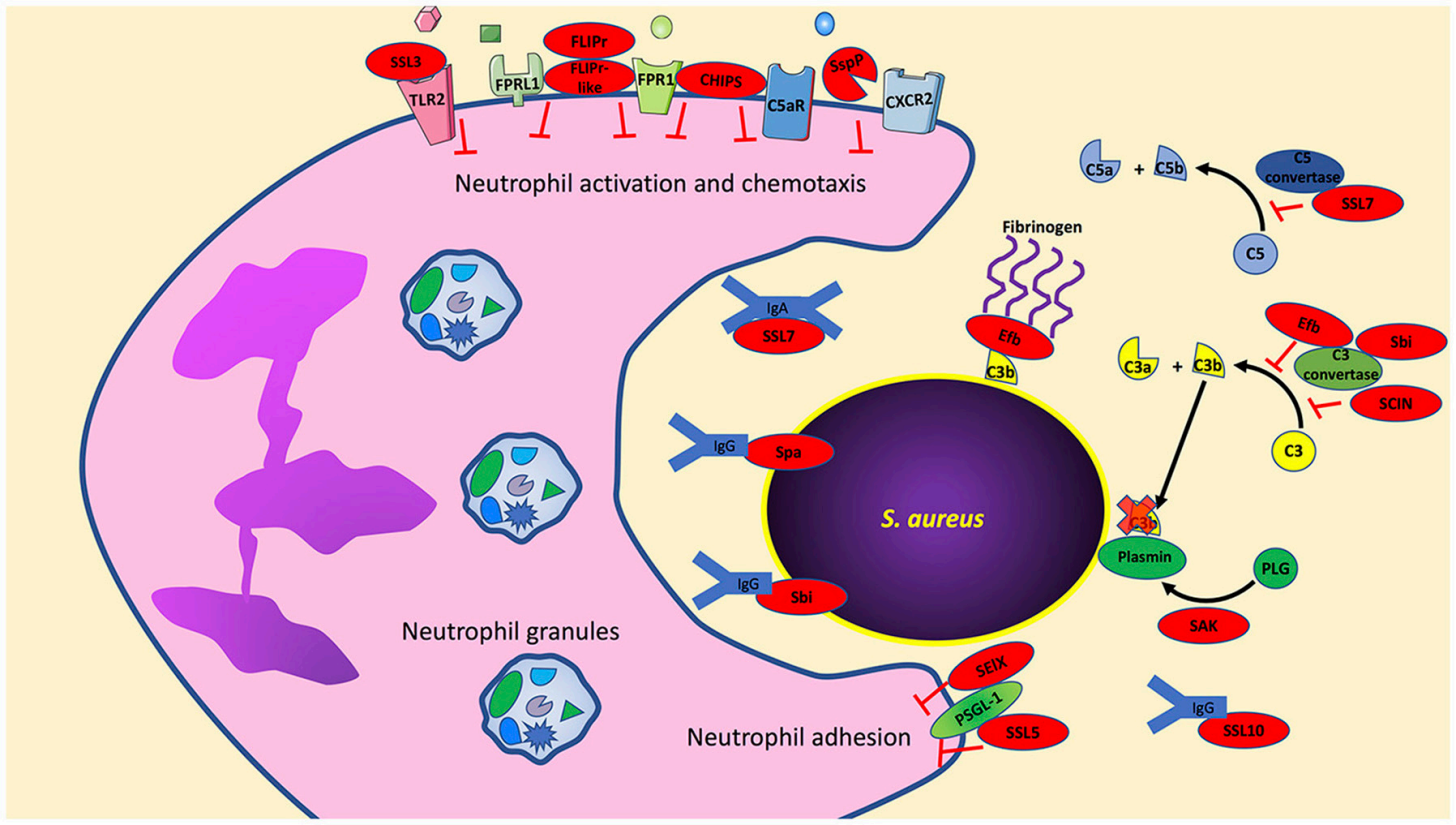
*S. aureus* has an arsenal of virulence factors to inhibit neutrophil activation, chemotaxis, and phagocytosis. Multiple virulence factors produced by *S. aureus* target key host effector proteins, for example, Efb, Sbi, and SCIN target the complement protein C3 convertase to prevent formation of C3a and C3b. Further studies are needed to determine if the production of these virulence factors are truly redundant or if they have multiple host targets as is the case with Sbi, which also targets immunoglobulins. Bacterial components indicated in red. PLG, plasminogen; SAK, staphylokinase; SspP, staphopain A.

In contrast to our understanding of how *S. aureus* may impact priming, there is an abundance of data that highlight how specific *S. aureus* factors impact neutrophil activation, chemotaxis and adhesion. In this section, we highlight individual virulence factors that have been studied for their ability to disrupt these key mechanisms (Figure 1).

## CHIPS

The chemotaxis inhibitory protein of *S. aureus* (CHIPS) is a 14.1-kDa exoprotein that inhibits neutrophil migration and activation (De Haas et al., 2004). CHIPS prevents neutrophils from responding to both host- and bacteria-derived chemoattractants. Neutrophil activation by host-derived C5a is inhibited by CHIPS binding to the C5a receptor (C5aR). Similarly, neutrophil detection of bacteria-derived formylated peptides is inhibited by CHIPS binding to the formyl peptide receptor (FPR). CHIPS shows high affinity to both C5aR and FPR with *K*_*D*_-values of 1.1 ± 0.2 and 35.4 ± 7.7 nM, respectively; the affinity of C5aR and FPR for their natural ligands is within the CHIPS binding affinity to C5aR and FPR (Falk et al., 1982; Huey and Hugli, 1985; Postma et al., 2004). While C5aR and FPR are G-protein coupled receptors (GPCRs), the active blocking domain in CHIPS is distinct for inhibiting C5aR and FPR (Haas et al., 2005). By using FITC-labeled CHIPS, Postma et al. demonstrated that CHIPS is not internalized following binding to neutrophil C5aR and FPR and its activity is ATP and cell-signaling independent (Postma et al., 2004). It has also been demonstrated that CHIPS binds to C5aR and FPR extracellularly and does not act as an agonist since receptor binding does not induce calcium mobilization (De Haas et al., 2004). It was further demonstrated that intravenously administered CHIPS was able to inhibit mouse neutrophil influx following intraperitoneal administration of C5a despite a 30-fold lower affinity of CHIPS for mouse C5aR (De Haas et al., 2004). Finally, neutralizing antibodies to CHIPS have been observed in human sera isolated from both normal donor controls and *S. aureus* infected samples, implying CHIPS plays an active role during staphylococcal infection (Wright et al., 2007).

## FLIPr and FLIPr-like

Neutrophils express formyl peptide receptor-like-1 (FPRL1). This receptor is activated by diverse peptides and proteins including: the synthetic peptides Trp-Lys-Tyr-Met-Val-D-Met- NH2 (WKYMVm), and L-conformer WKYMVM, lipoxin A_4_, the T21/DP107 leucine zipper-like domain of the HIV-1 envelope protein gp41, serum amyloid A, the mitochondrial peptide fragment MYFINILTL, the antimicrobial peptide LL-37, and prion peptide fragments (Fiore et al., 1994; Su et al., 1999a,b; Chiang et al., 2000; De Yang et al., 2000; Christophe et al., 2001; Le et al., 2001). Activation of FPRL1 on neutrophils leads to intracellular calcium mobilization and chemotaxis, while superoxide production through FPRL1 is ligand dependent (Bae et al., 2003). *S. aureus* produces two proteins that bind FPRL1 and inhibit its activation. FPRL1 inhibitory protein (FLIPr) is a secreted protein that binds and antagonizes both FPRL1 and FPR, but FPRL1 antagonism by FLIPr is much stronger than on FPR (Prat et al., 2006). Similarly, a second FPRL1 antagonist termed FLIPr-like shares 73% overall homology with FLIPr (Prat et al., 2009). FLIPr and FLIPr-like show similar inhibitory activity toward FPRL1 following stimulation with the FPRL1 agonist MMK-1; however, FLIPr-like antagonism toward FPR is 100-fold stronger than that of FLIPr following stimulation with fMLF (Prat et al., 2009). As expected, neutrophil exposure to purified FLIPr and FLIPr-like completely inhibits neutrophil chemotaxis toward the FPRL1 agonist MMK-1 (Prat et al., 2006, 2009).

FLIPr and FLIPr-like have also been shown to bind different FcγR isoforms to block IgG binding (Stemerding et al., 2013). Stemerding et al. showed that FLIPr preferentially binds to FcγR class II receptors while FLIPr-like can bind to FcγR cla ss I, II, and III receptors. As expected, neutrophil pretreatment with FLIPr or FLIPr-like significantly reduced phagocytosis of *S. aureus* opsonized with purified IgG (Stemerding et al., 2013). However, it should be noted that phagocytic inhibition of neutrophils toward *S. aureus* opsonized with human serum by FLIPr and FLIPr-like was only observed at low serum concentrations (< 1% serum). At higher serum concentrations (1–10% serum), FLIPr and FLIPr-like do not inhibit neutrophil-mediated phagocytosis of opsonized *S. aureus*. Under these higher serum conditions, neutrophil phagocytosis of opsonized *S. aureus* is likely mediated by complement receptors, which are not antagonized by FLIPr and FLIPr-like proteins. It follows that experiments using neutrophils treated with FLIPr and FLIPr-like had reduced uptake of *S. aureus* only when opsonized with complement inactivated serum thus showing the specific inhibition of FLIPr and FLIPr-like toward serum immunoglobulins (Stemerding et al., 2013).

## Staphopain A

The CXCR2 chemokine receptor (also known as IL8RB) is highly expressed on human neutrophils and has high specificity for the potent neutrophil chemoattractants CXCL1, CXCL2, and CXCL7, as well as CXCL8 (IL-8, which can also bind to CXCR1) (Yoshimura et al., 1987; Baggiolini et al., 1989; Ben-Baruch et al., 1997; Ritzman et al., 2010). *S. aureus* infection induces CXCL1, CXCL2, CXCL8 production resulting in neutrophil recruitment (Mempel et al., 2003; Sasaki et al., 2003; Olaru and Jensen, 2010). To prevent neutrophil recruitment induced by CXCR2 ligands, *S. aureus* secretes the cysteine protease staphopain A which cleaves the N-terminus of CXCR2 required for ligand binding (Laarman et al., 2012). Neutrophils pretreated with staphopain A blocked calcium mobilization following stimulation with CXCL1 and CXCL7. Importantly, these chemokines only bind CXCR2 (and not CXCR1). In contrast, staphopain A did not inhibit neutrophil calcium mobilization following stimulation with CXCL8 since it can also signal through CXCR1. The impacts of staphopain A *in vivo* are lacking since staphopain A is specific for human CXCR2 and does not cleave murine CXCR2 (Laarman et al., 2012).

## SElX and SSL5

Neutrophils express P-selectin glycoprotein ligand 1 (PSGL-1) on the cellular surface to bind P- and E-selectin on activated endothelial cells or platelets and L-selectin expressed on leukocytes (Moore et al., 1995; Guyer et al., 1996; Hidalgo et al., 2007; Huo and Xia, 2009; Stadtmann et al., 2013). PSGL-1 binding to selectins tethers neutrophils to activated endothelial cells and is a first step in the process of transmigration. *S. aureus* secretes two proteins that inhibit PSGL-1 binding to selectins. The staphylococcal enterotoxin-like toxin X (SElX) is a highly conserved superantigen that binds glycosylated PSGL-1 on neutrophils and inhibits binding to P-selectin (Wilson et al., 2011; Fevre et al., 2014). Similarly, the staphylococcal superantigen-like 5 (SSL5) protein directly binds PSGL-1 on neutrophils inhibiting rolling on endothelial cells (Bestebroer et al., 2007). In addition to binding PSGL-1, SSL5 also binds to other GPCRs but only inhibits the ligands that require the N-terminal domain of their respective receptors for activation (Bestebroer et al., 2009). Thus, SSL5 pretreatment of neutrophils inhibited activation induced by C3a, C5a, CXCL1, and CXCL8 (Bestebroer et al., 2009).

## SSL3

Toll-like receptor 2 (TLR2) recognizes staphylococcal peptidoglycan-associated lipoproteins (Fournier and Philpott, 2005; Kurokawa et al., 2009). TLR2 can also discriminate between diacylated and triacylated lipoproteins by associating with TLR6 or TLR1, respectively (Takeuchi et al., 2001, 2002). The recognition of staphylococcal lipoproteins such as the staphylococcal iron transporter C (SitC) are exclusively dependent on TLR2 to induce immune activation (Stoll et al., 2005; Kurokawa et al., 2009; Müller et al., 2010). The importance of TLR2 during *S. aureus* infection is highlighted by TLR2^−/−^ mice that show increased susceptibility and nasal colonization to *S. aureus* compared to wild-type mice (Takeuchi et al., 2000; González-Zorn et al., 2005; Hoebe et al., 2005). In addition, TLR2^−/−^ mice infected with *S. aureus* show alterations in cytokines that modulate neutrophil function including TNF-α and IL-1β (Knuefermann et al., 2004). Engagement of TLR2 influences many neutrophil functions including: adhesion molecule expression, reactive oxygen species production (following stimulation with fMLF), and modulates CXCL8, and chemokine receptor expression (Sabroe et al., 2003). *S. aureus* produces the staphylococcal superantigen-like 3 protein that binds TLR2 to inhibit activation in neutrophils and other cell types expressing TLR2 (Bardoel et al., 2012; Yokoyama et al., 2012). Crystal structures showed that SSL3 binding to TLR2 reduced the lipopeptide binding pocket by ~50%, which inhibited binding of the TLR2 agonist Pam_2_CSK_4_ (Koymans et al., 2015). The same study demonstrated SSL3 can bind a preformed TLR2- Pam_2_CSK_4_ complex and this blocked TLR2-TLR1 and TLR2-TLR6 heterodimerization thereby inhibiting downstream signaling.

## Strategies used by *S. aureus* to inhibit neutrophil phagocytosis

Phagocytosis is a process by which neutrophils, and other phagocytes, ingest particles from their extracellular environment including bacteria and host cells. In neutrophils, this sequestration results in the formation of an intracellular compartment termed the phagosome following invagination of the cellular membrane. Neutrophil granules fuse with the phagosome to release antimicrobial peptides and proteins, as well as produce reactive oxygen species to kill invading microorganisms. Neutrophils bind to pathogen-associated molecular patterns on the *S. aureus* surface to initiate the phagocytic process. Furthermore, binding of immunoglobulins to *S. aureus* and complement activation enhances phagocytosis, as well as engages different neutrophil receptors resulting in branching downstream signaling. *S. aureus* employs strategies and produces a wide range of virulence factors to disrupt neutrophil phagocytosis. Herein, we will highlight select strategies used by *S. aureus* to disrupt neutrophil phagocytosis. Since a complete review on the strategies used by *S. aureus* to avoid phagocytosis is outside the scope of this publication, we recommend previous detailed reviews focusing on immunoglobulin and complement evasion by *S. aureus* to inhibit phagocytosis (Lambris et al., 2008; Serruto et al., 2010; van Kesse et al., 2014).

## Capsule synthesis

The production of capsular polysaccharide by *S. aureus* has been proposed as an antiphagocytic evasion strategy but its actual role in inhibiting neutrophil phagocytosis remains controversial. Indeed, previous studies have shown that *S. aureus* strains producing high levels of capsular polysaccharide withstand neutrophil killing better than microencapsulated strains (Xu et al., 1992; Thakker et al., 1998). However, it should be noted that under these experimental conditions neutrophil killing of *S. aureus* was correlated to the ability of neutrophils to associate with bacteria without clearly showing ingestion. Furthermore, the experimental conditions used suspended instead of adherent neutrophils which can greatly influence neutrophil ingestion of *S. aureus* (Lu et al., 2014). Also, an intraperitoneal *S. aureus* infection model showed no differences in neutrophil intracellular staphylococcal survival between a highly and microencapsulated strain (Gresham et al., 2000). Notably, the predominant clinical isolate USA300 is unencapsulated, further questioning the role of capsule synthesis in *S. aureus* pathogenicity (Montgomery et al., 2008; Carrel et al., 2015).

## Complement inhibition

Human pathogens have developed strategies to evade complement, thus inhibiting immune recognition, cytokine production, and neutrophil uptake (Lambris et al., 2008; Serruto et al., 2010). *S. aureus* produces several virulence factors that target different machinery in the complement system. The staphylococcal complement inhibitor (SCIN), is a 9.8-kDa exoprotein that specifically binds and inhibits human C3 convertases (Rooijakkers et al., 2005a, 2006). SCIN binding to C3 convertases does not prevent C3 binding but completely inhibits generation of C3b and thus opsonization of *S. aureus* by C3b (Rooijakkers et al., 2005a; Ricklin et al., 2009; Garcia et al., 2010). In addition, SCIN stabilizes C3 convertases by competing with factor H which accelerates decay of C3 convertases (Ricklin et al., 2009). *In vitro*, recombinant SCIN can significantly inhibit neutrophil phagocytosis of human serum opsonized *S. aureus* resulting in increased bacterial survival (Rooijakkers et al., 2005a). *In vivo* studies with SCIN are lacking since it is human specific, but SCIN is immunogenic since a limited study with 80 healthy people and 20 individuals with recurring staphylococcal infections showed all produced antibodies to SCIN (Rooijakkers et al., 2005a).

*S. aureus* is also equipped with virulence factors that target complement without direct binding to C3 convertase. Staphylokinase is a secreted protein that binds human plasminogen converting it into its active form plasmin (Parry et al., 2000; Mölkänen et al., 2002; Rooijakkers et al., 2005b). Plasmin, a serine protease, is bound to the *S. aureus* surface and degrades C3 convertase-dependent C3b to prevent deposition on the bacterial surface. Human neutrophils show decreased phagocytic activity toward human serum opsonized *S. aureus* (depleted of IgG and IgM to rule out immunoglobulin mediated phagocytosis) and pretreated with recombinant staphylokinase (Rooijakkers et al., 2005b). In addition, *S. aureus* secretes the extracellular fibrinogen-binding (Efb) protein that binds to C3 to prevent cleavage to C3b and can also directly bind C3b deposited on the extracellular bacterial membrane (Lee et al., 2004; Ko et al., 2013). As its name implies, the extracellular fibrinogen-binding protein also contains a fibrinogen binding domain that recruits fibrinogen and inhibits neutrophil phagocytosis (Ko et al., 2013). Complement receptor recognition of C3b is blocked by a thick layer of fibrinogen linked by Efb on the bacterial surface (Ko et al., 2013). *Ex vivo* studies with human blood exposed to Efb and *S. aureus* showed a significant reduction in neutrophil phagocytosis compared to *S. aureus* alone. Similarly, supernatants from wild-type *S. aureus* reduced neutrophil phagocytosis of opsonized *S. aureus* compared to supernatants from Efb-deficient *S. aureus* in the presence of fibrinogen. Finally, neutrophils from mice intraperitoneally infected with GFP-expressing wild-type *S. aureus* or an Efb-deficient mutant showed significantly higher ingestion of the Efb mutant compared to the wild-type (Ko et al., 2013). While this observation suggests that sufficient levels of Efb are produced by *S. aureus* to have an *in vivo* effect, it should be noted that intraperitoneal injection was performed with *S. aureus* grown to late exponential phase without washing culture toxins. Thus, increased and unknown levels of Efb were likely present from the beginning giving an advantage to *S. aureus* to prevent neutrophil phagocytosis.

## Inhibition of immunoglobulin recognition

Complement and immunoglobulins function as opsonins to enhance *S. aureus* recognition and ingestion by neutrophils. While *S. aureus* produces virulence factors that inhibit complement activation and deposition on the bacterial surface, the efficacy of these virulence factors *in vivo* is controversial since immunoglobulin opsonization plays a redundant role in opsonophagocytosis. In fact, opsonophagocytosis assays investigating the role of *S. aureus* virulence factors that disrupt complement must be done in the absence of immunoglobulins to observe an inhibitory effect on phagocytosis because *S. aureus* produces virulence factors that inhibit opsonization by immunoglobulins. Protein A, encoded by the *spa* gene, is one of the better characterized virulence factors produced by *S. aureus* that inhibits neutrophil phagocytosis. Protein A is a 42-kDa secreted and membrane bound protein that binds the constant Fcγ region of IgG thus preventing proper engagement of Fcγ receptors on neutrophils and antigen recognition (Forsgren and Sjöquist, 1966; Kronvall et al., 1970; Sjödahl, 1977). Indeed, early studies clearly showed that purified protein A inhibited neutrophil phagocytosis of *S. aureus* independent of capsule polysaccharide on the bacterial surface (Dossett et al., 1969; King and Wilkinson, 1981). More recent studies have demonstrated protein A induces B cell proliferation and production of V_H_3 serum IgG and IgM (Pauli et al., 2014; Kim et al., 2016). Although abundant, these antibodies do not provide protection against *S. aureus*. Generation of protein A variants that were unable to bind immunoglobulin induce a protective antibody response presumably via allowing Fc mediated uptake of opsonized *S. aureus* (Falugi et al., 2013). These data suggest that mechanisms of neutrophil recognition and uptake of *S. aureus* are likely critical to resolution of infection.

*S. aureus* produces a second binding protein of immunoglobulin (termed Sbi) that also binds IgG to inhibit neutrophil phagocytosis (Jacobsson and Frykberg, 1995; Zhang et al., 1998, 1999; Smith et al., 2011). Human neutrophils can phagocytose significantly more opsonized Sbi-deficient *S. aureus* compared to wild-type, and Sbi plays a protective role in *ex vivo* whole human blood killing assays (Smith et al., 2011). Interestingly, Sbi binds to lipoteichoic acid in the bacterial cell surface to remain anchored, while it can also exist in a secreted form that binds complement protein C3 to induce its degradation away from the bacterial surface (Burman et al., 2008; Upadhyay et al., 2008). Sbi also plays another immunomodulatory role that was demonstrated to impact neutrophil function. In a mouse model of peritonitis Sbi induced production of IL-6 and CXCL-1, which resulted in neutrophil recruitment and subsequently an exacerbated inflammatory response (Gonzalez et al., 2015).

In addition to Sbi, *S. aureus* secretes other proteins that bind both immunoglobulins and complement proteins to disrupt neutrophil phagocytosis. The 23-kDa staphylococcal superantigen-like 7 (SSL7) exoprotein binds to human IgA with high affinity (*K*_*D*_ of 1.1 nM) and inhibits FcαR binding on the neutrophil surface (Langley et al., 2005). Using an *ex vivo* whole blood infection model it was demonstrated that binding of IgA by SSL7 reduced neutrophil phagocytosis of *S. aureus* (Bestebroer et al., 2010). SSL7 also binds to the C5 complement protein (*K*_*D*_ of 18 nM) and inhibits C5a generation in an IgA binding-dependent manner, as well as formation of the C5b-9 membrane attack complex (Langley et al., 2005; Bestebroer et al., 2010; Laursen et al., 2010; Lorenz et al., 2013). *In vivo* mouse studies showed that administration of purified SSL7 inhibited neutrophil recruitment following exposure to heat-killed *S. aureus*. Thus, SSL7 serves diverse functions that include inhibiting neutrophil phagocytosis, recruitment, and complement activation (Bestebroer et al., 2010; Lorenz et al., 2013). Another staphylococcal superantigen-like protein termed SSL10 also binds to IgG (*K*_*D*_ of 220 nM) and to the complement protein C1q (Itoh et al., 2010). SSL10 significantly inhibited neutrophil ingestion of IgG-opsonized *S. aureus* by blocking binding of FcγR (Patel et al., 2010).

Taken together, it is clear that *S. aureus* produces virulence factors that decrease neutrophil phagocytosis via production of physical barriers (capsule polysaccharide), inhibition of complement activation leading to decreased deposition of opsonins, and disruption of immunoglobulin binding to bacterial antigens. However, the efficacy of these virulence factors has been difficult to determine. Neutrophil-mediated phagocytosis of *S. aureus* is efficient in the absence of opsonins. *In vitro* studies have shown that adherent neutrophils rapidly ingest both opsonized and unopsonized *S. aureus* (Lu et al., 2014). Thus, neutrophils contain pattern-recognition receptors that are sufficient for phagocytosis of *S. aureus* independent of opsonins. Clearly, the redundancy in neutrophil receptor-mediated phagocytosis mechanisms compounds studies and strongly suggests the combination of *S. aureus* virulence factors are needed to thwart the innate ability of neutrophils to recognize this pathogen.

## Evasion strategies used by *S. aureus* to survive neutrophil killing

**Figure 2:**
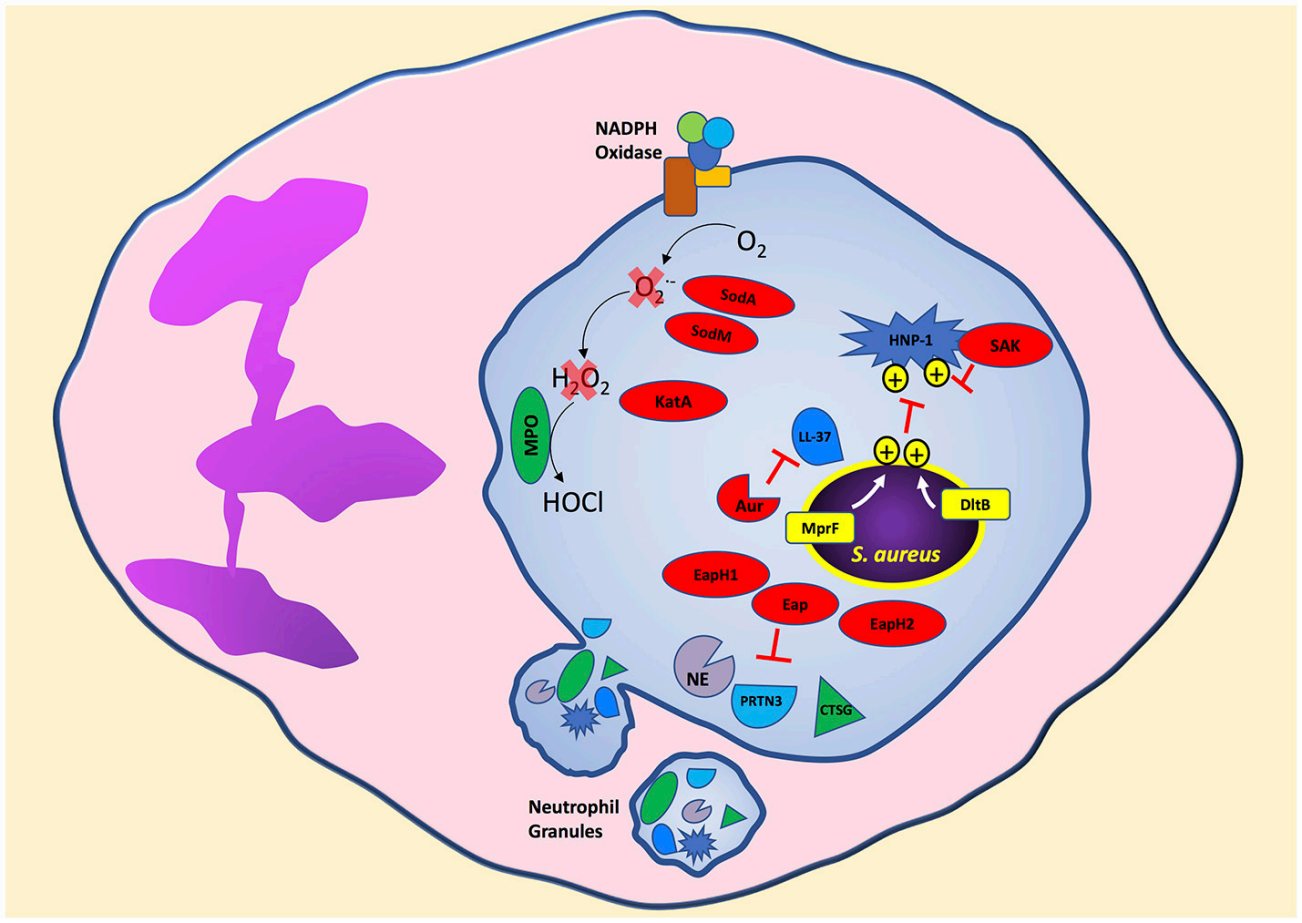
*S. aureus* produces virulence factors that target different neutrophil bactericidal mechanisms following phagocytosis. Cationic antimicrobial peptides are ineffective toward *S. aureus* due to the presence of positive charges on the bacterial surface transferred by MprF and DltB. *S. aureus* secretes virulence factors that degrade antimicrobial proteins and enzymes released into the neutrophil phagosome. In addition, neutrophil reactive oxygen species production is decreased by *S. aureus* virulence factors that degrade intermediate reactive oxygen species like superoxide and hydrogen peroxide to reduce the formation of the highly bactericidal chemical agent hypochlorous acid. Bacterial components indicated in red. Aur, aureolysin; PRTN3, proteinase 3; CTSG, cathepsin G; SAK, staphylokinase.

*S. aureus* is clearly decorated to avoid ingestion by neutrophils. Additionally, *S. aureus* is loaded with mechanisms to disarm potent neutrophil bactericidal mechanisms. Soon after initiation of the phagocytic process, and possibly before complete sealing of the phagosome, neutrophil granules mobilize to fuse and release antimicrobial agents into the phagosome containing *S. aureus* (Flannagan et al., 2009). Following phagocytosis, neutrophils expose *S. aureus* to antimicrobial peptides, proteins, and reactive oxygen species to degrade essential bacterial proteins and disrupt homeostasis resulting in bacterial death (Figure 2). In this section, we will highlight strategies used by *S. aureus* to avoid oxidative and non-oxidative mechanisms of neutrophil killing. Although we separate *S. aureus* evasion of oxidative and non-oxidative killing, it is important to remember that these mechanisms likely have synergistic antimicrobial activity in a physiological setting.

## *S. aureus* evasion of non-oxidative killing

The *dlt* operon in *S. aureus*, containing genes *dltABCD* is involved in activation and transfer of D-alanine, and increases tolerance to cationic antimicrobial peptides (AMPs), including human neutrophil peptide-1 (HNP-1, also known as alpha-defensin), by incorporation of positively charged D-alanine into teichoic acids (Peschel et al., 1999; Collins et al., 2002). Teichoic acids are highly negatively charged by deprotonized phosphate groups that electrostatically interact with cationic peptides. Thus, a *S. aureus dlt* mutant strain retains a negatively charged teichoic acid backbone and is significantly more susceptible to killing by cationic peptides (Peschel et al., 1999). *In vitro, S. aureus dlt* mutants are highly susceptible to neutrophil non-oxidative killing mechanisms whereas wild-type *S. aureus* is resistant and mainly susceptible to neutrophil oxidative killing (Collins et al., 2002). The importance of the *dlt* operon to increase *S. aureus* survival was observed in an *in vivo* mouse model of sepsis, which showed increased host mortality infected with wild-type *S. aureus* compared to the non-lethal *S. aureus dlt* mutant (Collins et al., 2002). Similarly, the *mprF* gene in *S. aureus* encoding lysylphosphatidylglycerol (LPG) synthetase confers resistance to cationic AMPs by transferring the positively charged L-lysine to the negatively charged lipid phosphatidylglycerol creating LPG (Peschel et al., 2001; Oku et al., 2004; Ernst et al., 2009). Electrostatic repulsion between cationic AMPs and the attached lysine prevents binding of cationic AMPs and disruption of the bacterial membrane. Wild-type *S. aureus* is more resistant to killing by human neutrophils compared to a *S. aureus mprF* mutant (Peschel et al., 2001). Thus, *S. aureus* modifies its cell surface as an evasion strategy by increasing electrostatic repulsion of neutrophil antimicrobial peptides leading to decreased bacterial killing.

In addition to cell membrane modifications to decrease efficacy of antimicrobial peptides by electrostatic repulsion, *S. aureus* produces proteins that directly bind, inhibit, and degrade antimicrobial peptides. Staphylokinase, which activates plasminogen to the active serine protease plasmin leading to degradation of IgG and C3b (Rooijakkers et al., 2005b), also binds HNP-1 and inhibits its bactericidal activity (Jin et al., 2004). The cathelicidin LL-37, which has potent staphylococcal bactericidal activity, is degraded by the *S. aureus* secreted metalloprotenaise aureolysin (Sieprawska-Lupa et al., 2004). Thus, *S. aureus* strains producing aureolysin are significantly more resistant to LL-37 than aureolysin-negative strains. Furthermore, the extracellular adherence protein (Eap) and the Eap-homologs 1 and 2 (EapH1 and EapH2) are neutrophil serine protease inhibitors (Harraghy et al., 2005). Purified Eap, EapH1, or EapH2 inhibit the activity of neutrophil elastase, proteinase 3, and cathepsin G which are found in neutrophil azurophilic granules and are bactericidal to *S. aureus* (Papayannopoulos et al., 2010; Stapels et al., 2014).

## *S. aureus* evasion of oxidative killing

Neutrophil activation leads to assembly of the NADPH oxidase resulting in production of reactive oxygen species (ROS; DeLeo and Quinn, 1996; DeLeo et al., 1999; Bréchard and Tschirhart, 2008; Nunes et al., 2013). Assembly of the membrane and cytoplasmic NADPH oxidase components results in electrons shuttled from NADPH to oxygen producing superoxide. Spontaneous and myeloperoxidase (MPO) catalyzed dismutation of superoxide produces hydrogen peroxide, which in the presence of MPO and chloride results in the highly bactericidal agent hypochlorous acid/hypochlorite anion (Kettle et al., 2007; Klebanoff et al., 2013). Neutrophil ingestion exposes *S. aureus* to concentrated ROS in the phagosome resulting in oxidation and chlorination of bacterial and host proteins (Green et al., 2014). Inhibition of neutrophil ROS production by diphenyleneiodonium significantly increases *S. aureus* survival following neutrophil phagocytosis even with functioning non-oxidative killing mechanisms (Hampton and Winterbourn, 1995; Hampton et al., 1996). Individuals with chronic granulomatous disease carry mutations in components of NADPH oxidase preventing formation of superoxide and hydrogen peroxide and suffer from recurring bacterial infections including *S. aureus* (Lekstrom-Himes and Gallin, 2000; Assari, 2006). Thus, uninhibited neutrophil ROS production is essential for *S. aureus* clearance.

Bacterial pathogens have developed strategies to inhibit ROS killing (Flannagan et al., 2009). *S. aureus* produces several enzymes in response to neutrophils to degrade and detoxify ROS. The ROS chain of production begins with the formation of superoxide from oxygen. *S. aureus* produces two superoxide dismutases, encoded by *sodA* and *sodM*, that convert superoxide into hydrogen peroxide and molecular oxygen (Karavolos et al., 2003). Superoxide dismutase activity assessed on nondenaturing polyacrylamide gel electrophoresis from the *S. aureus* cytoplasm shows three distinct bands consisting of SodA and SodM homodimers, and a SodA-SodM hybrid (Clements et al., 1999; Valderas and Hart, 2001). Wild-type *S. aureus* is more resistant to superoxide stress compared to isogenic *sodA*, or *sodM*, or *sodAsodM*-deficient strains (Karavolos et al., 2003). In a mouse subcutaneous model of infection, significantly higher wild-type *S. aureus* colony forming units were recovered from the site of infection compared to single or double *sod* mutant strains suggesting a role for superoxide dismutase in virulence (Karavolos et al., 2003). The expression of *sodA* and *sodM* is increased under oxidative stress and regulated by the transcriptional regulator SarA (Ballal and Manna, 2009). Interestingly, *S. aureus* uses multiple regulatory systems to respond to oxidative stress. KatA, the only catalase encoded by *S. aureus* that degrades hydrogen peroxide to water and oxygen, is co-regulated by the ferric uptake repressor (Fur) and the peroxide response regulator (Horsburgh et al., 2001b). As expected, *katA* deficient *S. aureus* is more sensitive to killing by hydrogen peroxide compared to wild-type *S. aureus* (Horsburgh et al., 2001a). Furthermore, the alkyl hydroperoxide reductase (AhpC) increases *S. aureus* resistance to the organic-hydroperoxide cumene hydroperoxide and its absence increases *S. aureus* resistance to hydrogen peroxide by a compensatory increase in *katA* expression (Cosgrove et al., 2007). However, the significance of KatA in surviving neutrophil ROS production is unclear since studies have shown no significant differences in neutrophil killing of wild-type *S. aureus* compared to an isogenic *katA* deficient strain (Cosgrove et al., 2007).

Superoxide dismutase and catalase protect *S. aureus* from ROS through enzymatic degradation. *S. aureus* also produces virulence factors that inhibit ROS killing by different mechanisms. The iconic yellow-golden pigment of *S. aureus* is a result of the antioxidant carotenoid named staphyloxanthin and synthesized by genes encoding *crtM* and *crtN* (Wieland et al., 1994; Pelz et al., 2005). The production of staphyloxanthin increases *S. aureus* resistance to killing by hydrogen peroxide and singlet oxygen (Liu et al., 2005). In addition, staphyloxanthin increases *S. aureus* survival during exposure to neutrophil ROS; in contrast, staphyloxanthin does not confer protection during exposure to neutrophils from patients with chronic granulomatous disease or neutrophils pretreated with diphenyleneiodonium which have deficiencies in ROS production (Liu et al., 2005). Following exposure to neutrophil ROS, surviving bacteria undergo repair of oxidized proteins to maintain homeostasis. *S. aureus* encodes four methionine sulfoxide reductases that play a role in reducing oxidized methionine residues (Singh, 2003; Schöneich, 2005; Singh et al., 2015). Deletion of the methionine sulfoxide reductases *msrA1* and *msrB* increases *S. aureus* susceptibility to exogenous hydrogen peroxide and hypochlorous acid. Furthermore, a *msrA1* and *msrB* double mutant strain of *S. aureus* is more susceptible to neutrophil killing compared to wild-type (Pang et al., 2014). Finally, the SaeR/S TCS also plays a role in regulating virulence factors that decrease neutrophil hydrogen peroxide and hypochlorous acid production following *S. aureus* phagocytosis (Guerra et al., 2016). The SaeR/S-regulated virulence factors that decrease neutrophil ROS remain unknown and are an active area of research, but are independent of superoxide dismutase and catalase activity since their expression is not regulated by SaeR/S (Rogasch et al., 2006; Voyich et al., 2009; Nygaard et al., 2010; Sun et al., 2010).

Human neutrophils extrude DNA decorated with antimicrobial proteins termed neutrophil extracellular traps (NETs) to ensnare and kill bacteria as a terminal cell fate pathway that has been named NETosis and is dependent on ROS (Brinkmann et al., 2004; Fuchs et al., 2007; Galluzzi et al., 2012). Neutrophil exposure to *S. aureus* is a potent inducer of NETs (Pilsczek et al., 2010). However, *S. aureus* escapes NETs by secreting nuclease (*nuc*), an SaeR/S-regulated factor (Berends et al., 2010; Olson et al., 2013). Furthermore, the nuclease products from DNA degradation 2′-deoxyadenosine-3′-monophosphate and 2′-deoxyadenosine-5′-monophosphate are converted by the *S. aureus* exoprotein adenosine synthase (*adsA*) into 2′-deoxyadenosine (dAdo), which induces caspase-3-mediated apoptosis in macrophages (Thammavongsa et al., 2013). A murine intravenous infection model showed macrophages are unable to diffuse into kidney abscesses containing neutrophils and wild-type *S. aureus*; however, infection with a *nuc* or *adsA* deficient *S. aureus* strain allowed macrophages to efficiently infiltrate the neutrophil abscess (Thammavongsa et al., 2013).

## *S. aureus* modulation of neutrophil fate

Neutrophils undergo an apoptotic differentiation program in response to bacterial pathogens to limit host damage caused by a prolonged inflammatory response (Kobayashi et al., 2003). For excellent reviews on the mechanisms of neutrophil apoptosis and how bacterial pathogens modulate neutrophil fate, we recommend (Kennedy and Deleo, 2009; Rigby and DeLeo, 2012; Kobayashi et al., 2017). *S. aureus* produces pore-forming toxins that lyse neutrophils, other leukocytes, and red blood cells and this lytic activity promotes an intense inflammatory response. While these toxins clearly play a role in lysing neutrophils and other immune cells, their role if any beyond neutrophil cytolysis is unknown. Notable exceptions include Panton-Valentine Leukocidin (PVL) and LukGH which have been shown to prime neutrophils at sublytic concentrations and promote neutrophil extracellular trap formation, respectively (Graves et al., 2012; Malachowa et al., 2013). In addition, neutrophils exposed to *S. aureus* have been shown to undergo cell death with intact phagosomes undermining the role of cytolytic toxins in *S. aureus* escape from neutrophils (Kobayashi et al., 2010). For reviews on the lytic properties of these toxins and their targets, please refer to (DuMont and Torres, 2014; Reyes-Robles and Torres, 2016). Herein, we will highlight recent studies suggesting *S. aureus* modulates neutrophil fate to disrupt proper neutrophil clearance following phagocytosis of *S. aureus* independent of lytic activity.

Following phagocytosis, neutrophils are ingested by macrophages in a process called efferocytosis to remove spent neutrophils and limit inflammation (Martin et al., 2014). *S. aureus* inhibits macrophage efferocytosis of *S. aureus* laden neutrophils by upregulating the “don't eat me” signal CD47 (Greenlee-Wacker et al., 2014). Interestingly, macrophage production of cytokines that modulate neutrophil fate was altered and inflammasome activation was reduced in response to neutrophils harboring *S. aureus* compared to *S. aureus* alone. *In vitro*, human neutrophils containing *S. aureus* do not undergo the classical apoptotic death pathway as they fail to activate caspase-3, as well as caspase-2, -8, and -9. Instead, neutrophil death in this *in vitro* model in response to *S. aureus* is dependent on receptor-interacting protein 1 (RIP-1) which is a hallmark of programmed cell death or necroptosis. Studies by Zurek et al. showed that the SaeR/S TCS plays a role in modulating neutrophil fate by inhibiting IL-8 production and NF-κB activation (Greenlee-Wacker et al., 2014; Zurek et al., 2015). Programmed neutrophil death in response to wild-type *S. aureus* was accelerated compared to exposure to a *saeR/S* deletion mutant.

In addition, ROS production is implicated in programmed neutrophil death leading to NET formation (NETosis) and as discussed above *S. aureus* inhibits neutrophil ROS production (Fuchs et al., 2007; Akong-Moore et al., 2012). Interestingly, Pilsczek et al. described a NET forming mechanism that did not require neutrophil lysis and is ROS independent. Instead, DNA-containing vesicles budding from the nuclear membrane are released while maintaining neutrophil plasma membrane integrity (Pilsczek et al., 2010). Thus, these anuclear neutrophils may still retain phagocytic and chemotactic activity, as previously observed (Malawista et al., 1989). Further studies are needed to determine if inhibition of neutrophil ROS production by *S. aureus* modulates NETosis and to better elucidate ROS independent and dependent NET formation and NETosis. However, it is clear that *S. aureus* alters the neutrophil death program through a variety of mechanisms that impact not only cellular signaling by the neutrophil but clearance via efferocytosis.

## The other side of the equation: the ability of *S. aureus* to sense neutrophils

**Figure 3:**
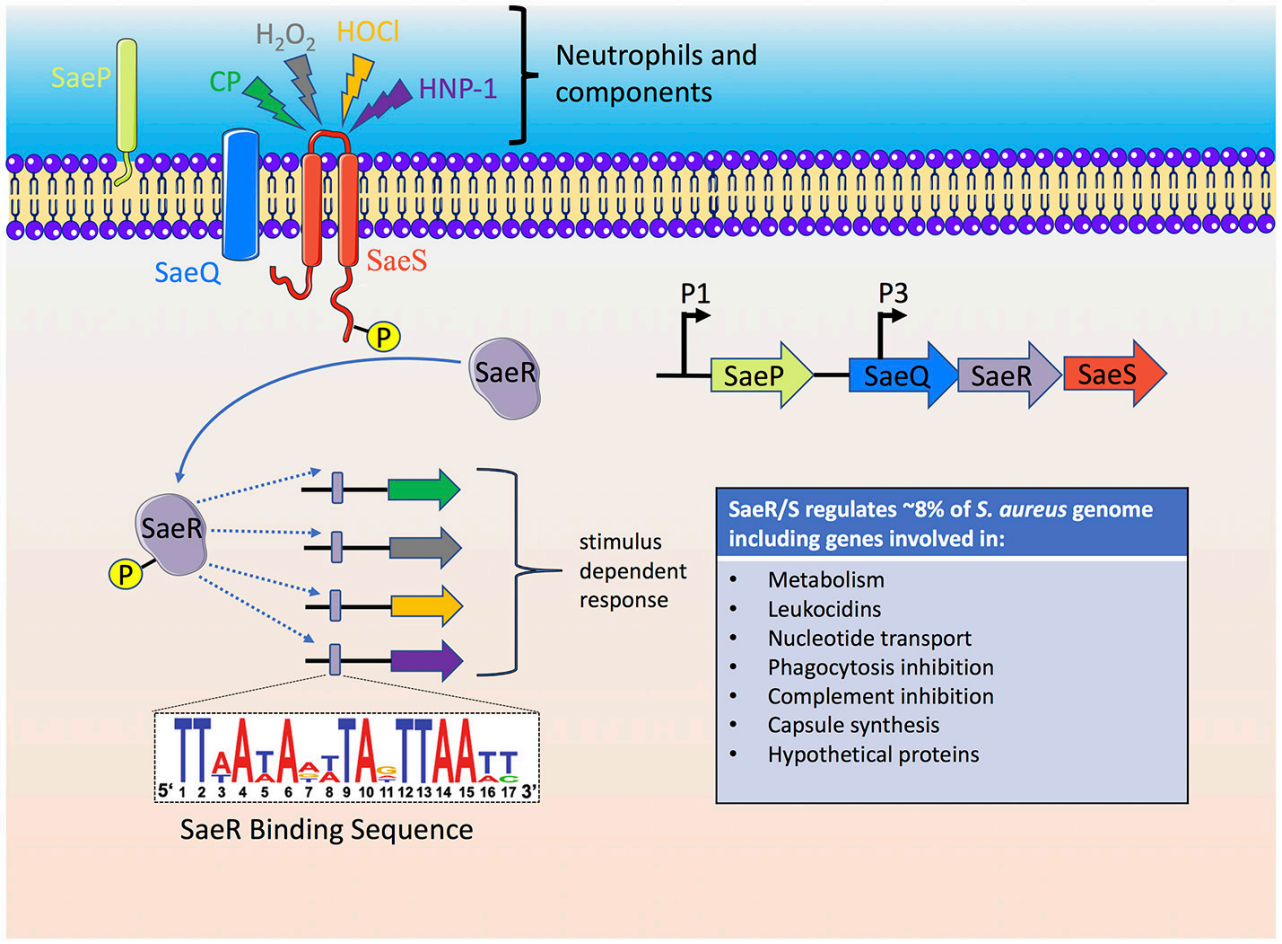
The *S. aureus* SaeR/S two-component system senses neutrophils and neutrophil components to activate an anti-host immune response. Activation of the SaeS histidine kinase results in autophosphorylation which is subsequently transferred to the SaeR response regulator. A promoter binding region recognized by phosphorylated SaeR (SaeR binding sequence) initiates gene transcription of SaeR/S-regulated virulence factors that target neutrophil antimicrobial mechanisms, cell fate and cellular signaling. The immediate genes transcribed by SaeR/S are dependent on the stimulus, i.e., the SaeR/S transcriptional profile is dynamic and specific to the stimulus. Since bacterial sensory-regulatory systems activate multiple virulence factors, therapeutic approaches to inhibit bacterial sensing, and activation is an active area of research. CP, calprotectin.

As reviewed above, the neutrophil has many mechanisms to sense and respond to *S. aureus* and likewise individual *S. aureus* factors have evolved to disrupt every step of the neutrophil response to the invading pathogen. However, the ability of *S. aureus* to sense the host has been underestimated. The *S. aureus* genome consists of 16 two-component systems (TCS) that sense environmental stimuli and regulate gene expression accordingly (Kawada-Matsuo et al., 2011). Of these 16 TCSs, SaeR/S is recognized as a major contributor to *S. aureus* pathogenesis and neutrophil evasion (Voyich et al., 2009; Figure 3). Geiger et al. was the first to demonstrate that the TCS SaeR/S contained an upstream promoter element that could be activated by human neutrophil products H_2_O_2_ and alpha-defensin (Geiger et al., 2008). SaeR/S target genes are differentially regulated in response to whole blood, neutrophils and neutrophil components (H_2_O_2_, alpha-defensin, and calprotectin; Voyich et al., 2005; Palazzolo-Ballance et al., 2007; Malachowa et al., 2011; Flack et al., 2014; Zurek et al., 2014; Cho et al., 2015). The nine amino acid extracellular loop of SaeS plays an important role in sensing different neutrophil-derived stimuli (Flack et al., 2014; Liu et al., 2015). Highly conserved residues M31, W32, and F33 on SaeS are essential to the appropriate activation of *sae*-target genes. Residue M31 is essential to the activation of *sae* regulon and plays an important role in sensing neutrophil-derived stimuli including HNP-1. Strains with mutations in aromatic residues W32 and F33 have disrupted normal basal signaling of SaeS in the absence of inducing signals, yet both mutant kinases have appropriate activation of effector genes following exposure to neutrophil-derived stimuli (Flack et al., 2014). This posits that response regulators turn-on only subsets of genes based on the host stimulus. Such observations support a unique hypothesis that emerging strains of bacteria are not more virulent because they harbor new virulence factors but are better able to sense and respond to their human hosts.

SaeR/S is the best studied for its role in sensing the neutrophil but additional studies are needed to understand just how *S. aureus* senses the neutrophil *in vivo*. Certainly other regulatory systems play a role either directly or potentially by responding to the activation of SaeR/S, however, these studies are currently limited. Considering HNP-1 is recognized early following phagocytosis, the recognition of HNP-1 is likely very important for *S. aureus* adaptation to the neutrophil environment. Interestingly, the recognition of HNP-1 by SaeS has not been demonstrated to influence the genes in *S. aureus* directly correlated with combating the antimicrobial capacity of antimicrobial peptides [e.g., *dlt* and *mprF* are regulated by the GraRS two-component system (Yang et al., 2012)] but instead appears to ready *S. aureus* for combating neutrophil reactive oxygen species and for producing cytolytic toxins. Additionally, the GraRS system recognizes cationic peptides including LL-37, and polymyxin B but does not recognize HNP-1 implying a complex interplay of sensory systems to combat the neutrophil (Yang et al., 2012). Another interesting two-component gene regulatory system is WalK/R. Studies have demonstrated this system is essential for *S. aureus* viability (Dubrac and Msadek, 2004; Dubrac et al., 2007). However, studies using a strain of *S. aureus* with a constitutively active WalR response regulator demonstrated constitutive expression caused up-regulation of virulence genes that are known to be SaeR-regulated including *coa, hla hlgA, hlgB, hlgC*, and *sbi* (Delauné et al., 2012). Although the exact mechanism of communication between WalR and SaeR/S is unknown data suggest WalR influences the SaeR/S system indirectly. This is inferred since deletion of *saeR/S* in a strain that constitutively expressed WalR showed the impact of WalR on the virulence factors during growth *in vitro* was through SaeR/S (Delauné et al., 2012). However, in mouse models of infection constitutive expression of WalR in *S. aureus* caused increased neutrophil recruitment and enhanced bacterial clearance compared to wild-type *S. aureus*. Constitutive expression of WalR decreasing virulence *in vivo* was independent of SaeR/S and linked to WalR dependent peptidoglycan release promoting an inflammatory environment that recruited neutrophils (Delaune et al). Currently the stimulus recognized by WalK is unknown. Understanding what stimulus(i) activates this system may reveal how WalK/R and SaeR/S regulation of virulence are linked. Additionally, the fatty acid kinase VfrB, that lacks any traditional DNA-binding domain, has been shown to influence gene expression of well-defined SaeR/S-dependent genes (Bose et al., 2014; Krute et al., 2016). However, when a *vfrB* mutant strain was exposed to the known SaeS-activation stimulus HNP-1, this kinase no longer impacted virulence genes regulated by SaeR/S (Krute et al., 2016). These data further emphasize the importance of defining virulence regulation in physiologically relevant environments and highlight the complexity of virulence gene regulation in *S. aureus*.

## The challenge of investigating neutrophil—*S. aureus* interactions *In vivo*

Investigating the neutrophil-*S. aureus* interactions *in vivo* has been challenging. The relevance of using the murine immune system to mimic interactions between *S. aureus* and the human immune system is heavily debated (Buer and Balling, 2003; Kim et al., 2014; Montgomery et al., 2014; Reizner et al., 2014; Tseng et al., 2015). Interestingly, though HNP-1 is recognized as the most prominent trigger of *saeR/S*, murine neutrophils are deficient in its production (Inaba et al., 2010). Not only do they differ in the production of HNP-1 but notable differences are observed in chemokine production, cell trafficking, and susceptibility to secreted toxins (Johnston et al., 1990; Singer and Sansonetti, 2004; Parker and Prince, 2012; Tseng et al., 2015). Often the importance of single *S. aureus* virulence genes is highlighted in models of infection; however, canonical *S. aureus* toxins such as HlgA, HlgC, and LukF-PVL have extremely limited lytic activity on murine cells (Liu, 2009; Baba-Moussa et al., 2011; Parker and Prince, 2012; Vandenesch et al., 2012). While deficiencies in the murine model are apparent it remains the most prominent and cost effective method to explore relationships *in vivo* between *S. aureus* and the host immune system. To that end, advances are being made toward creating more effective murine models that retain benefits such as availability of working with immune-deficient lines while displaying similar disease characteristics as observed in human hosts (Montgomery et al., 2014; Tseng et al., 2015). Tseng et al. developed a “humanized” murine model wherein NOD/SCID/IL2ry^null^ mice are engrafted with human CD34^+^ umbilical cord blood cells leading to the production of human immune cells within mice (Tseng et al., 2015). These mice demonstrated susceptibility to *S. aureus* infections at concentrations similar to what is observed in humans as well as increased susceptibility to LukF-PVL. In future efforts, humanized murine models such as this may be key to understanding how *S. aureus* regulates its pathogenesis in the context of a more relevant host environment and may be critical to advance research into effective therapeutics.

## Potential of therapeutics to augment neutrophil function against *S. aureus*

Vaccine development to prevent or reduce *S. aureus* infections has not been successful (Fowler and Proctor, 2014). Antibody based vaccines targeting individual virulence factors have not conferred significant protection against *S. aureus*, and in some cases have even increased mortality (Fowler et al., 2013). As discussed above, *S. aureus* produces virulence factors that target specific neutrophil receptors to inhibit neutrophil functions. However, *S. aureus* virulence factors show redundancy in the neutrophil functions they inhibit. For example, multiple virulence factors target the complement pathway and immunoglobulin recognition to inhibit neutrophil phagocytosis and neutralizing one of these virulence factors is not likely to have a significant effect *in vivo*. Thus, vaccine approaches that simultaneously target multiple *S. aureus* virulence factors are needed and encouragingly some are in the pipeline (Torre et al., 2015; Frenck et al., 2016).

Targeting *S. aureus* sensory/regulatory systems has great potential for therapeutic and vaccine development. Theoretically, chemical inhibitors and neutralizing antibodies of sensory/regulatory systems could inhibit *S. aureus* from sensing its environment and producing virulence factors that disrupt host antimicrobial functions. The SaeR/S TCS is an attractive candidate for vaccine development since SaeR/S-regulated factors disrupt key neutrophil functions that include production of ROS, cytokine expression, and cell fate (Watkins et al., 2011, 2013; Nygaard et al., 2013; Zurek et al., 2014; Guerra et al., 2016). Many of the best studied immunomodulatory factors and cytolytic proteins that target neutrophil function are regulated by SaeR/S including Sbi, Efb, HlgABC, Hla, LukG/H (LukA/B), and PVL (Nygaard et al., 2010). Thus, inhibiting SaeS from sensing its environment has the potential to prevent expression of multiple virulence factors in response to host signals. In this line of thinking, targeting *S. aureus* quorum sensing via inhibition of Agr has been proposed as a therapeutic approach to inhibit production of virulence of factors (Clatworthy et al., 2007; Park et al., 2007; Cegelski et al., 2008; Kaufmann et al., 2008; Njoroge and Sperandio, 2009; Rutherford and Bassler, 2012). For a review on the *S. aureus* Agr regulatory system please refer to (Painter et al., 2014). In brief, findings have identified synthetic peptides, small molecules, and antibodies that disrupt *S. aureus* Agr-dependent quorum sensing either by blocking the sensor histidine kinase AgrC from recognizing its cognate signal autoinducing peptide (AIP), by inhibiting the AgrA response regulator from binding DNA, or by antibody interference of AIP (Park et al., 2007; Kaufmann et al., 2008; Kirchdoerfer et al., 2011; Tal-Gan et al., 2013; Broderick et al., 2014; Murray et al., 2014; Sully et al., 2014). Using this approach, reductions in mouse dermonecrosis following infection with *S. aureus*, increased bacterial clearance, and reduced hemolytic activity have been reported (Tal-Gan et al., 2013; Sully et al., 2014). In addition, the development of materials with quorum sensing inhibitors to apply during a skin infection is an active area of research and showing promising results (Broderick et al., 2014; Kratochvil et al., 2017). However, studies are needed to fully elucidate the effects of inhibiting sensory/regulatory systems since this can also increase the production of negatively regulated virulence factors and the impact on cross talk between regulatory systems that might result in compensation by one system at the loss of another, is not known.

Modulation of the inflammatory response may be another attractive target for therapeutics that might increase the likelihood of an effective neutrophil response. However, additional research is needed to identify key inflammatory mediators that resolve *S. aureus* infection and to understand those that exacerbate disease. Studies have identified IL-17 as a key determinant of proper host defense during *S. aureus* cutaneous infection (Cho et al., 2010). Murphy et al. identified γδ T cells as the major source of IL-17 during peritonitis caused by *S. aureus* and identified a subset of memory γδ T cells that enhanced IL-17 production to protect the host during subsequent *S. aureus* infection (Murphy et al., 2014). Thus, immunotherapies that enhance host bactericidal functions by neutralizing detrimental inflammatory responses, increasing beneficial cytokines at the site of infection, or stimulating the expansion of protective T cell subpopulations in response to *S. aureus* show therapeutic potential. Further research is also needed to understand site-specific cytokine requirements to mount an effective host response to *S. aureus* infection.

Lastly, as discussed above, the use of mouse models has undeniably increased our understanding of *S. aureus*-host interactions. However, due to the specificity of many of the *S. aureus* virulence factors toward human cells, improved models of disease are needed like the “humanized” mouse as discussed above. With improved models and advances in understanding neutrophil-*S. aureus* interactions, perhaps future reviews will be able to document neutrophils as the clear winner in this epic immune battle.

In conclusion, the ability of *S. aureus* to survive a neutrophil encounter is thought to contribute significantly to the virulence of this pathogen. This is exemplified by the observed increase in susceptibility to *S. aureus* infections in individuals suffering from defects that alter normal neutrophil function, such as chronic granulomatous disease, leukocyte adhesion deficiency, and neutropenia (Bodey et al., 1966; Pincus et al., 1976; Dale et al., 1979; Lekstrom-Himes and Gallin, 2000). It is clear that neutrophil antimicrobial activity is essential to eliminate *S. aureus*. However, studies have also demonstrated that increased neutrophil numbers at the site of infection can exacerbate disease (Gresham et al., 2000; McLoughlin et al., 2008) and that modulation of the neutrophil inflammatory response can significantly impact outcome of infection (Watkins et al., 2013; Zurek et al., 2015). Clearly more studies are needed to define the neutrophil responses that resolve a *S. aureus* infection. Finding the right balance of controlling inflammation while maintaining an effective neutrophil antimicrobial response will be key to the design of an effective therapeutic.

## Author contributions

FG and JV wrote, edited, prepared manuscript. TB, DP, and ES wrote sections and contributed equally.

### Conflict of interest statement

The authors declare that the research was conducted in the absence of any commercial or financial relationships that could be construed as a potential conflict of interest.
